# The Prevalence, Usage Patterns, and Complications of Contact Lens Use Among University Students in Damascus, Syria: A Cross‐Sectional Cohort Study

**DOI:** 10.1002/hsr2.71041

**Published:** 2025-07-14

**Authors:** Fares Kahal, Sedra Al‐Habal, Saeed Kadri, Omar Helwani, André Torbey

**Affiliations:** ^1^ Faculty of Medicine Syrian Private University Damascus Syria

**Keywords:** contact lenses, ocular complications, ophthalmology, Syria, university students

## Abstract

**Background:**

Contact lenses are widely used globally, primarily for vision correction and cosmetic enhancement. However, improper handling can lead to many complications. Understanding wearer practices and risk factors is crucial to prevent these adverse effects. This study aims to assess the prevalence of contact lens (CL) wearers among Syrian Private University students, their cleaning and care practices, and potential risk factors linked to complications and symptoms.

**Methods and Materials:**

This cross‐sectional study was conducted at the Syrian Private University (SPU) from December 2022 to April 2023. Data was collected from 500 students who met the inclusion and exclusion criteria. Data on demographics, hygiene practices, and complications were collected via a paper‐based questionnaire. Statistical analysis was performed using SPSS 25.0, with significance set at *p* < 0.05.

**Results:**

Participants were predominantly females (88.8%) with a mean age of 22.41 years. Most participants used contact lenses without a valid prescription (64.6%) for aesthetic reasons (44.3%). The main vision problems were myopia (38.9%), astigmatism (10.3%), and hyperopia (4%). Complications included red eye (68%), dacryorrhea (63%), dry eye (58.4%), and blurred vision (43%). Factors significantly associated with various ocular complications include showering or swimming while wearing contact lenses, sharing lenses, exceeding the recommended renewal period, and improper or inconsistent use of contact lens solutions.

**Conclusion:**

Our findings indicate that improper contact lens use and non‐prescribed acquisitions are prevalent among Syrian university students, driven largely by cosmetic motives. These behaviors contribute to preventable ocular complications, underscoring a critical public health concern. The results highlight the need for regulatory measures, targeted education, and clinical interventions to promote safe practices, reduce complications, and safeguard eye health within the population.

## 1 Introduction

Contact lenses (CL) are thin, transparent ocular devices that delicately adhere to the corneal surface [1]. Notably, in 1888, Adolf Eugen Fick, a German physiologist, fitted the first glass contact lens on patients. His invention is often regarded as the first truly practical contact lens [2]. Over the recent decades, the use of CL has grown rapidly worldwide, appealing to individuals of all ages, from children to the elderly. This widespread adoption, estimated to include approximately 140 million users globally, reflects significant accessibility [3].

Soft CL, in particular, has emerged as the most commonly prescribed option globally [4]. This preference is reflected in numerous studies, one of which, a cross‐sectional study conducted at King's Abdulaziz University, Jeddah, Saudi Arabia, revealed that a significant 80.2% of CL wearers opt for the comfort of soft lenses [5].

CL serves a variety of purposes beyond simple vision correction, with applications in optical, therapeutic, diagnostic, and cosmetic fields. Optically, they are used to correct high myopia, anisometropia, and conditions like keratoconus and irregular astigmatism. Therapeutically, they aid in the management of corneal pathologies, dry eyes, and post‐keratoplasty care. They can also be used for diagnostic purposes, such as in gonioscopy and fundus examination. Cosmetic applications include addressing conditions like corneal scars and phthisis bulbi. Additionally, contact lenses serve preventive functions, such as protecting against exposure keratitis, and are used in occupational settings, like for sports professionals, police officers, and pilots [6].

However, in spite of their manifold uses and advantages, frequent and improper usage of CL may cause a spectrum of complications. These complications range from mild issues, such as epithelial edema, typically reversible, to more severe conditions like microbial keratitis, a potentially vision‐threatening infection caused by organisms such as Pseudomonas aeruginosa and Acanthamoeba. Beyond these examples, many other potential complications, including allergic reactions, conjunctival changes, and lens deposits, highlight the importance of proper care, regular follow‐ups, and adherence to safe contact lens practices to minimize risks [6, 7].

Given the limited research on contact lens (CL) usage in Syria, particularly among university students, this study aims to fill this gap by assessing the prevalence of CL wearers at Syrian Private University and evaluating their cleaning and care practices. Additionally, the study seeks to identify potential risk factors for complications and symptoms associated with improper CL use. The findings from this study will contribute to raising awareness of the importance of proper CL care, and to the reduction of associated risks and complications.

## 2 Methods and Materials

### 2.1 Study Design, Setting, and Participants

This cross‐sectional study was conducted at the Syrian Private University (SPU) from December 2022 to April 2023. A total of 1,700 students were initially approached to identify contact lens wearers. After applying the inclusion and exclusion criteria, a final sample of 500 students was selected for the study.

### 2.2 Inclusion Criteria

The study included Syrian Private University students of all ages from the faculties of medicine, dentistry, pharmacy, engineering, business administration, and computer technology, who wore contact lenses on an almost daily basis.

### 2.3 Exclusion Criteria

Students who did not wear contact lenses regularly, as well as those who were pregnant or immunosuppressed, were excluded from the study. As a result, 1,200 students did not meet the eligibility criteria.

### 2.4 Study Population and Sampling Method

A convenience sampling method was used to initially approach 1,700 students from the faculties of medicine, dentistry, pharmacy, engineering, business administration, and computer technology. From this group, 500 students who met the inclusion criteria were selected for the study.

### 2.5 Sample Size Calculation

SPU has a student population of approximately 5,000. Sample size calculation determined that 357 participants would be statistically sufficient to achieve a 95% confidence level and a 5% margin of error based on this population size. This ensured that the study was adequately powered to detect significant associations.

### 2.6 Pilot Testing and Instrument Validation

Before the main data collection, a pilot study was conducted with 50 students to assess the validity and reliability of the survey instrument. The pilot yielded a satisfactory Cronbach's alpha score ranging from 0.68 to 0.74, indicating acceptable internal consistency.

### 2.7 Informed Consent and Ethical Approval

Informed consent was obtained from all study participants after clearly explaining the study's objectives, the use of collected data, and the confidentiality of their responses. This study received approval from the Syrian Private University Ethics Committee. Although no approval number was provided, the study was conducted with the full endorsement of the committee and in adherence to ethical guidelines.

### 2.8 Questionnaire Administration

Participants were given paper‐based anonymous questionnaires. To minimize potential misunderstanding, the data collection team offered brief explanations of any medical terminology included in the survey. The questionnaire consisted of three sections:

- **Section 1:** Collected demographic and personal information including age, gender, family income, employment status, smoking status, and medical conditions.
- **Section 2:** Focused on hygienic practices related to contact lens use as reported by the participants.
- **Section 3:** Documented contact lens‐related complications reported by the students.

The full questionnaire is available in the Appendix.

### 2.9 Statistical Analysis

Data analysis was performed using the Statistical Package for Social Sciences (SPSS) for Windows, Version 25.0. Categorical variables were reported using frequencies and percentages, and continuous variables were reported using mean and standard deviation. The association between different hygienic practices and various complications was determined using the Chi‐square test, with a *p* of < 0.05 considered statistically significant.

### 2.10 Study Design

**Figure 1:**
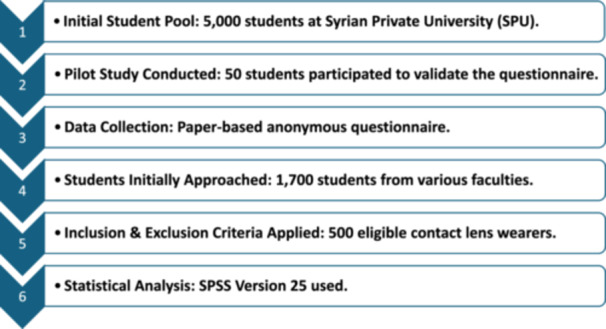
Study Design Flowchart.

Figure 1 provides a visual representation of the overall study design.

## 3 Results

In this study, the research cohort comprised 500 participants, with 56 (11.2%) males and 444 (88.8%) females, who had an average age of 22.41 ± 3.11 years. The majority of participants were enrolled in health sciences faculties (58.4%). Participants' detailed demographics and characteristics are presented in Table 1.

**Table 1:** Demographics characteristics of participants.

Age, mean (standard deviation)	22.41 (± 3.11)
Gender	*N* (%)
Male	56 (11.2)
Female	444 (88.8)
Smoking	
Yes	167 (33.4)
Negative smoking	117 (23.4)
No	216 (43.2)
Family income	
Very good	150 (30.0)
Good	296 (59.2)
Moderate	54 (10.8)
Working	
Yes	96 (19.2)
No	404 (80.8)
Faculty	
Business	93 (18.6)
Pharmacy	88 (17.6)
Medicine	131 (26.2)
Engineering	73 (14.6)
Dentistry	73 (14.6)
Information technology	42 (8.4)
Do you have any of the following medical conditions	
Rheumatoid diseases	15 (3.0)
Diabetes	8 (1.6)
Sjogren's syndrome	1 (0.2)
Crohn's disease	2 (0.4)
Polycystic ovaries	1 (0.2)

A significant portion of the student population, consisting of 323 individuals (64.6%), was found to have used CLs without a valid prescription. The most prevalent reason for wearing CLs among the students was for aesthetic purposes, as reported by 244 individuals (44.3%). Other causes for CLs usage were related to visual acuity issues, with myopia being the most prevalent (reported by 214 students, 38.9%), followed by astigmatism (57 students, 10.3%), and hyperopia (22 students, 4.4%).

In our study, it was observed that 63.6% of the participants did not adhere to the recommended renewal period for their CLs. Specifically, 38.8% consistently exceeded this period, while 24.8% did so occasionally. Regarding lens cleaning habits, only 0.4% of users admitted to never cleaning their lenses, whereas 96.4% reported cleaning them with contact lens solution. Approximately 5.4% of users reported sharing their CLswith others. Concerning water exposure, 16% of users exposed their lenses to water during showering, and 9% while swimming. Table 2 Represents the usage patterns, types of lenses, and practices among the participants.

**Table 2:** Usage patterns, types, and practices among contact lens users.

Did an ophthalmologist/optometrist prescribe lenses for you?	*N* (%)
Yes	177 (35.4)
No	323 (64.6)
Do you use lenses in	
Both eyes	489 (97.8)
One eye	11 (2.2)
How long have you been using lenses?	
Less than a month	48 (9.6)
More than a month and less than a year	85 (17)
More than a year and less than 3 years	146 (29.2)
More than 3 years	221 (44.2)
Reasons for using contact lenses	
Keratoconus	8 (1.45)
Astigmatism	57 (10.3)
After surgery	4 (0.7)
Cosmetic reasons	244 (44.3)
Myopia	214 (38.9)
Corneal abrasion	1 (0.2)
Hyperopia	22 (4.0)
Type of lens	
Rigid	17 (3.4)
Soft	285 (57)
Colored	177 (35.4)
Semi‐rigid	9 (1.8)
Do not know	12 (2.4)
Renewing contact lenses	
Daily	86 (17.2)
From week to month (wearable during sleep)	51 (10.2)
From week to month (unwearable during sleep)	210 (42)
3 months	5 (1.0)
6 months	17 (3.4)
8 months	1 (0.2)
Annually	25 (5.0)
Exceeding the recommended period of renewal	
Yes	194 (38.8)
No	182 (36.4)
Sometimes	124 (24.8)
Cleaning contact lens	
With contact lenses solution	482 (96.4)
With water	5 (1.0)
Never	2 (0.4)
Do not know	11 (2.2)
Using soap when washing hands before using lenses	
Yes	396 (79.2)
No	46 (9.2)
Occasionally	58 (11.6)
Showering with lenses	
Yes	41 (8.2)
No	420 (84)
Occasionally	39 (7.8)
Swimming with lenses	
Yes	44 (8.8)
No	433 (86.6)
Occasionally	23 (4.6)
Sharing lenses	
Yes	20 (4.0)
No	473 (94.6)
Occasionally	7 (1.4)

The principal complications observed among contact lens wearers were red eyes (reported by 68.2% of cases), followed by dacryorrhea (63.4%), dry eyes (58.4%), and conjunctivitis (50.2%). Diagnoses of conjunctivitis were made in 25% of participants by medical doctors, resulting from various pathogenic origins such as allergies, bacteria, and viruses. Meibomianitis was the least common complication, affecting only 1.2% of the sample. Table 3. presents the participant‐reported contact lens complications.

**Table 3:** Reported contact lenses‐related complication(s) by students' users.

Dry eyes	*N* (%)
Yes	292 (58.4)
No	208 (41.6)
White purulent secretions	
Yes	85 (17.0)
No	415 (83.0)
Eyelid swelling	
Yes	99 (19.8)
No	401 (80.2)
Conjunctival redness	
Yes	178 (35.6)
No	322 (64.4)
Eyelid adhesion when waking up	
Yes	65 (13.0)
No	435 (87.0)
Watery secretions	
Yes	120 (24.0)
No	380 (76.0)
Conjunctivitis symptoms	
Yes	251 (50.2)
No	249 (49.8)
Painful vesicles on the eyelid	
Yes	14 (2.8)
No	486 (97.2)
Eye itch	
Yes	169 (33.8)
No	331 (66.2)
Red eye	
Yes	341 (68.2)
No	159 (31.8)
Pain	
Yes	204 (40.8)
No	296 (59.2)
Dacryorrhea	
Yes	317 (63.4)
No	183 (36.6)
Blurred vision	
Yes	215 (43.0)
No	285 (57.0)
Photophobia	
Yes	69 (13.8)
No	431 (86.2)
Corneal ulcer	
Yes	32 (6.4)
No	468 (63.6)
Chalazion	
Yes	18 (3.6)
No	482 (96.4)
Stye	
Yes	54 (10.8)
No	446 (89.2)
Meibomianitis	
Yes	6 (1.2)
No	494 (98.8)
Loss of visual acuity	
Yes	144 (28.8)
No	229 (45.8)
Do not know	127 (25.4)
Conjunctivitis (diagnosed by doctors)	
Allergic conjunctivitis	63 (12.6)
Bacterial conjunctivitis	42 (8.4)
Viral conjunctivitis	20 (4.0)
No	375 (75.0)
Keratitis (diagnosed by doctors)	
Bacterial keratitis	12 (2.4)
Fungal keratitis	3 (0.6)
Viral keratitis	4 (0.8)
No	481 (96.2)

Our study has unveiled significant associations between improper hygienic practices and various complications. Specifically, we found a statistically significant relationship between the incidence of dry eye and two factors: showering while wearing lenses (*p* = 0.009) and swimming with lenses (*p* = 0.006). Additionally, sharing CLs was found to be significantly linked to conjunctivitis (*p* = 0.003). Our analysis also revealed significant links between certain hygienic practices and the development of red eye and dacryorrhea among contact lens wearers. Notably, exceeding the recommended lens renewal period was associated with both conditions (*p* = 0.014 for red eye and *p* = 0.0038 for dacryorrhea), as was cleaning lenses with contact lens solution (*p* = 0.001 for red eye and *p* = 0.002 for dacryorrhea).“Table 4. Presents the associations between various wrong hygienic practices and associated complications.

**Table 4:** Associations between various wrong hygienic practices and associated complications.

Hygiene practices	Dry eyes		*p* value
	Yes	No	
Exceeding the recommended period of renewal			0.050
Yes	110 (60.44)	72 (39.56)	
No	101 (52.06)	93 (47.94)	
Sometimes	81 (65.32)	43 (34.68)	
Cleaning contact lens			0.085
With contact lenses solution	285 (59.13)	197 (40.87)	
With water	2 (40.0)	30 (60.0)	
Never	2 (100)	0 (0.00)	
Do not know	3 (27.27)	8 (72.73)	
Using soap when washing hands using lenses			0.273
Yes	233 (58.84)	163 (41.16)	
No	30 (65.22)	16 (34.78)	
Occasionally	29 (50.0)	29 (50.0)	
Showering with lenses			0.0098*
Yes	30 (73.17)	11 (26.83)	
No	233 (55.48)	187 (44.52)	
Occasionally	29 (74.36)	10 (25.64)	
Swimming with lenses			0.0065*
Yes	33 (75.0)	11 (25.0)	
No	241 (55.66)	192 (44.34)	
Occasionally	18 (78.26)	5 (21.74)	
Sharing lenses			0.590
Yes	13 (65.0)	7 (35.0)	
No	276 (58.35)	197 (41.65)	
Occasionally	3 (42.86)	4 (57.14)	

Hygiene practices	Conjunctivitis		*p* value
	Yes	No	
Exceeding the recommended period of renewal			0.771
Yes	88 (48.35)	94 (51.65)	
No	101 (52.06)	93 (47.94)	
Sometimes	62 (50.0)	62 (50.0)	
Cleaning contact lens			0.073
With contact lenses solution	247 (51.24)	235 (48.76)	
With water	2 (40.0)	3 (60.0)	
Never	0 (0.00)	2 (100)	
Do not know	2 (18.18)	9 (81.82)	
Using soap when washing hands using lenses			0.438
Yes	193 (48.74)	203 (51.26)	
No	26 (56.52)	20 (43.48)	
Occasionally	32 (55.17)	26 (44.83)	
Showering with lenses			0.172
Yes	26 (63.41)	15 (36.59)	
No	204 (48.57)	216 (51.43)	
Occasionally	21 (53.85)	18 (46.15)	
Swimming with lenses			0.448
Yes	26 (59.09)	18 (41.91)	
No	213 (49.19)	220 (50.81)	
Occasionally	12 (52.17)	11 (48.83)	
Sharing lenses			0.0036*
Yes	17 (85.0)	3 (15.0)	
No	232 (49.05)	241 (50.95)	
Occasionally	2 (28.57)	5 (71.43)	

Hygiene practices	Red eye		*p* value
	Yes	No	
Exceeding the recommended period of renewal			0.014*
Yes	114 (62.64)	68 (37.36)	
No	130 (67.0)	64 (33.0)	
Sometimes	97 (78.23)	27 (21.77)	
Cleaning contact lens			0.0017*
With contact lenses solution	336 (69.71)	146 (30.29)	
With water	2 (40.0)	3 (60.0)	
Never	0 (0.00)	2 (100)	
Do not know	3 (27.27)	8 (72.73)	
Using soap when washing hands using lenses			0.411
Yes	266 (67.17)	130 (32.83)	
No	31 (67.39)	15 (32.61)	
Occasionally	44 (75.86)	14 (24.14)	
Showering with lenses			0.536
Yes	25 (60.98)	16 (39.02)	
No	288 (68.57)	132 (31.43)	
Occasionally	28 (71.79)	11 (28.28)	
Swimming with lenses			0.416
Yes	27 (61.36)	17 (38.64)	
No	300 (69.28)	133 (30.72)	
Occasionally	14 (60.87)	9 (39.13)	
Sharing lenses			0.346
Yes	14 (70.0)	6 (30.0)	
No	324 (68.50)	149 (31.50)	
Occasionally	3 (42.86)	4 (57.14)	

Hygiene practices	Dacryorrhea		*p* value
	Yes	No	
Exceeding the recommended period of renewal			0.0038*
Yes	100 (54.95)	82 (45.05)	
No	126 (64.95)	68 (35.05)	
Sometimes	91 (73.39)	33 (26.61)	
Cleaning contact lens			0.0020*
With contact lenses solution	313 (64.94)	169 (35.06)	
With water	2 (40.0)	3 (60.0)	
Never	0 (0.00)	2 (100)	
Do not know	2 (18.18)	9 (81.82)	
Using soap when washing hands using lenses			0.310
Yes	247 (62.37)	149 (37.63)	
No	28 (60.87)	18 (39.13)	
Occasionally	42 (72.41)	16 (27.59)	
Showering with lenses			0.345
Yes	23 (56.10)	18 (43.90)	
No	266 (63.33)	154 (36.67)	
Occasionally	28 (71.79)	11 (28.21)	
Swimming with lenses			0.241
Yes	23 (52.27)	21 (47.73)	
No	278 (64.20)	155 (35.80)	
Occasionally	16 (79.57)	7 (30.43)	
Sharing lenses			0.863
Yes	12 (60.0)	8 (40.0)	
No	300 (63.42)	173 (36.58)	
Occasionally	5 (71.43)	2 (28.57)	

## 4 Discussion

This study is the first of its kind in Syria to the best of our knowledge, providing a comprehensive exploration of contact lens (CL) usage and complications. The findings hold significant implications, highlighting the need for healthcare professionals to address the prevalent use of CLs, particularly in the context of inadequate adherence to hygiene standards that may negatively impact users' quality of life. This concern is amplified by the lack of published studies on the epidemiology and consequences of CL use in Syria.

Our findings show that 88.8% of CL users in the study sample were female, reflecting a global trend likely driven by the increasing popularity of CLs for cosmetic purposes. Notably, 44.3% of participants identified cosmetic reasons as their primary motivation for wearing CLs, consistent with findings from other studies [5, 8, 9, 10]. The alignment between the high percentage of female CL users and their cosmetic motivations illustrates the significant influence of aesthetic preferences on CL usage trends. This relationship highlights how cosmetic appeal shapes demographic patterns in CL prescriptions, reflecting both local cultural norms and broader international trends in the adoption of CLs for enhancing personal appearance. As a result, there is an increase in CL usage without a prescription, with 64.6% of participants acquiring CLs without a proper prescription. This concerning trend in Syria, which is higher than the rates reported in neighboring countries (11%–38.7%), underscores the urgent need for regulatory measures similar to those enforced in developed nations [1, 11].

Although hygienic practices were largely satisfactory, certain behaviors and environmental exposures likely contributed to the reported complications. Specifically, activities like showering and swimming while wearing CLs were significantly linked to dry eyes. While safe for drinking, home tap water is not sterile and contains microorganisms that can contaminate CLs, potentially leading to serious complications such as keratitis [12]. For example, exposure to water is strongly associated with contact lens‐related illnesses, such as Acanthamoeba keratitis [13]. Similarly, even brief exposure to chlorinated pool water increases the risk of contact lens colonization by bacterial species present in the water, such as *Pseudomonas aeruginosa* [14]. Once colonized, these bacteria can compromise the ocular surface defenses through mechanisms such as tear stagnation or microtrauma, potentially leading to CL‐related keratitis [13]. Fortunately, keratitis emerged as one of the least frequent complications in this study, occurring in only 4% of cases, which is a low incidence likely attributed to the participants' correct hygiene practices, highlighting the pivotal role of adherence to recommended hygiene measures in minimizing such complications.

Exceeding the recommended replacement period for CLs is a key factor in various ocular complications, primarily due to an increased risk of microbial infections and discomfort. Research has shown that individuals who fail to adhere to replacement guidelines face an increased likelihood of conditions such as microbial keratitis and corneal abrasions [15, 16, 17]. This risk is further amplified by improper hygiene practices and extended use of lenses beyond their intended use period [18]. A survey in Saudi Arabia found that 43.8% of participants exceeded the recommended replacement period [19]. Similarly, studies in other regions reported even higher noncompliance rates, such as 65.1% in Spain [20]. In our study, 38.8% of participants did not adhere to replacement schedules, a behavior significantly associated with red eyes and dacryorrhea. A possible explanation is that overwearing CLs may cause them to act as a reservoir for microbes and irritants such as lipids, which can be absorbed from the environment and the tear film. This can lead to symptoms such as eye redness and tearing, and may contribute to other conditions like contact lens‐induced papillary conjunctivitis (CLPC) and microbial infections [13, 21, 22].

The use of CL solutions was significantly associated with the development of red eyes and dacryorrhea. CL solutions contain various chemical substances which may differ significantly between brands [23]. Some of these components can irritate the eyes, leading to symptoms such as redness and tearing [13]. Another possible explanation for these symptoms is improper use of the solutions, such as “topping off” old solution with new solution in the lens case or using an inadequate amount of solution [23]. These practices compromise the proper removal of irritants and effective disinfection of the lenses, increasing the risk of ocular complications. Additionally, regardless of how the solutions are used, certain ineffective formulations have been implicated in outbreak cases of microbial keratitis globally, highlighting the importance of selecting effective and safe solutions to minimize the risk of infection [24, 25, 26, 27].

This study on contact lens use among Syrian private university students underscores the need for a multifaceted approach to address critical findings. The predominance of female users highlights the importance of targeted health education campaigns to promote safe usage practices and raise awareness about the risks of acquiring lenses without a valid prescription. Additionally, the high rate of non‐prescribed lens acquisition calls for stricter regulatory oversight of CL distribution in Syria to ensure user safety.

Regular follow‐up appointments with optometrists, particularly for those using lenses for aesthetic purposes, should be encouraged. Pre‐approval tests based on medical history can further safeguard against complications. The observed associations between improper lens renewal practices, activities, and symptoms like red eyes and dacryorrhea provide valuable insights that healthcare professionals can use to offer tailored guidance.

During the literature search, it became evident that limited research exists on the changes that occur to contact lenses after their expiration and the complications associated with expired lenses. Similarly, research is sparse on the complications arising from the improper use of contact lens solutions. These gaps highlight the need for further studies to better understand these risks and develop evidence‐based recommendations.

The Department of Ophthalmology at the college should lead awareness initiatives, involving students in campaigns to educate the public about proper CL practices. Expanding this study to include a broader Syrian population will be crucial to validate the study's findings.

By integrating educational programs, enhanced regulations, and targeted healthcare interventions, such strategy aims to promote responsible CL use, minimize preventable ocular complications, and improve the understanding of CL practices and associated risks in Syria. Future research in Syria should investigate the role of demographic factors, such as age, sex, income, smoking, and education, as potential risk factors for contact lens‐related complications. This will help provide a more comprehensive understanding of the influence of these variables on the hygiene practices and health outcomes associated with contact lens use.

## 5 Conclusion

This study highlights significant issues with contact lens use among Syrian university students, including high rates of non‐prescribed acquisition and cosmetic reasons, especially among females. Improper hygiene practices, such as showering and swimming with lenses, were associated with dry eyes, sharing contact lenses with conjunctivitis, and exceeding replacement schedules with red eyes and dacryorrhea. Additionally, the use of CL solutions was significantly associated with red eyes and dacryorrhea. These findings underline the need for targeted health education on proper hygiene, solution use, and adherence to replacement schedules, as well as stricter regulations on CL distribution. Further research in a larger Syrian population is needed to confirm these results and improve ocular health practices.

## 6 Limitation

This study has several limitations. As a self‐reported questionnaire, responses were subject to recall bias and inaccuracies, and the predominance of female participants (89%) limits the generalizability of findings. The overrepresentation of medical sciences students may have skewed results, and the cross‐sectional design prevents establishing causation. Geographic limitation to Damascus universities may not reflect broader Syrian populations, and the self‐reported data could not verify practices like lens hygiene or prescription adherence, potentially underestimating complications. Variability in lens brands and solutions was not accounted for, and cultural or regulatory factors specific to Syria may limit applicability elsewhere. Future research should address these issues through longitudinal designs, diverse samples, and clinical verification of practices.

## Author Contributions

**Fares Kahal:** conceptualization, methodology, writing – review and editing, investigation, validation, visualization, project administration, supervision, writing – original draft. **Sedra Al‐Habal:** conceptualization, investigation, writing – original draft, methodology, writing – review and editing, visualization. **Saeed Kadri:** validation, formal analysis, writing – review and editing, writing – original draf, software. **Omar Helwani:** conceptualization, data curation, methodology, investigation. **André torbey:** supervision; validation.

## Ethics Statement

Written informed consent was obtained from every participant before participation. The Research Ethics Committee in the Syrian Private University approved the study protocol. All procedures performed in studies involving human participants were in accordance with the ethical standards of the institutional and/or national research committee and with the 1964 Helsinki declaration and its later amendments or comparable ethical standards. Due to the absence of an approval number, this study was conducted with the committee's full endorsement.

## Conflicts of Interest

The authors declare no conflicts of interest.

## 1 Transparency Statement

This study was conducted in accordance with ethical guidelines and received approval from the Research Ethics Committee at the Syrian Private University. The authors confirm that all relevant data, methods, and analyses have been transparently reported.

## Data Availability

The data that support the findings of this study are available from the corresponding author upon reasonable request.

## 1

**Survey**

1. **Do you use contact lenses?**
  o Yes
  o No
2. **Age:** (Open‐ended)
3. **Gender:**
  o a. Male
  o b. Female
4. **Smoking:**
  o a. Yes
  o b. Passive smoking
  o c. No
5. **Family income:**
  o a. Very good or Excellent
  o b. Good or Average
  o c. less than average
6. **Are you currently working?**
  o a. Yes
  o b. No
7. **If you are working, what is your field of work?** (Open‐ended)
8. **Faculty:**
  o a. Business
  o b. Pharmacy
  o c. Medicine
  o d. Engineering
  o e. Dentistry
  o f. Information Technology
  o g. Other: (Specify)
9. **Do you have any of these medical conditions?**
  o a. Rheumatoid diseases
  o b. Diabetes
  o c. Sjögren's syndrome
  o d. Crohn's disease
  o e. Polycystic ovary syndrome
  o f. None
10. **Did an ophthalmologist/optometrist prescribe lenses for you?**
  o a. Yes
  o b. No
11. **Do you use lenses in:**
  o a. Both eyes
  o b. One eye
12. **How long have you been using lenses?**
  o a. Less than a month
  o b. More than a month but less than a year
  o c. More than a year but less than 3 years
  o d. More than 3 years
13. **Reasons for using contact lenses:**
  o a. Keratoconus
  o b. Astigmatism
  o c. Post‐surgery
  o d. Cosmetic reasons
  o e. Myopia
  o f. Corneal abrasion
  o g. Hyperopia
  o h. Other: (Specify)
14. **If you have any of the conditions mentioned above, they are in:**
  o a. Both eyes
  o b. One eye
15. **Type of lenses:**
  o a. Rigid
  o b. Soft
  o c. Colored
  o d. Semi‐rigid
  o e. Do not know
16. **Renewing contact lenses:**
  o a. Daily
  o b. Weekly to monthly (wearable during sleep)
  o c. Weekly to monthly (non‐wearable during sleep)
  o d. Every 3 months
  o e. Every 6 months
  o f. Every 8 months
  o g. Annually
17. **Do you exceed the recommended renewal period?**
  o a. Yes
  o b. No
  o c. Sometimes
18. **How do you clean your contact lenses?**
  o a. With contact lens solution
  o b. With water
  o c. Never
  o d. Do not know
19. **Do you use soap when washing your hands before using lenses?**
  o a. Yes
  o b. No
  o c. Occasionally
20. **Do you shower with lenses on?**
  o a. Yes
  o b. No
  o c. Occasionally
21. **Do you swim with lenses on?**
  o a. Yes
  o b. No
  o c. Occasionally
22. **Do you share your contact lenses with anyone else?**
  o a. Yes
  o b. No
  o c. Maybe
23. **Do you experience dry eyes?**
  o **a. Yes**
  o **b. No**
24. **Have you been diagnosed by a doctor with any of the following?**
  o a. Corneal and conjunctival infections (yellow or gray discharge, eyelid swelling, pain, tearing, photophobia)
  o b. Eyelid margin inflammation (white or yellow crusts, loss of eyelashes)
  o c. Lacrimal secretion deficiency (dry eyes)
25. **Do you experience white purulent secretions?**
  o a. Yes
  o b. No
26. **Do you experience eyelid swelling?**
  o a. Yes
  o b. No
27. **Do you experience conjunctival redness?**
  o a. Yes
  o b. No
28. **Do your eyelids stick together when waking up?**
  o a. Yes
  o b. No
29. **Do you experience watery secretions?**
  o a. Yes
  o b. No
30. **Do you have symptoms of** conjunctivitis **(Redness, itching, tearing, discharge, and eye irritation.)?**
  o a. Yes
  o b. No
31. **Do you have painful** vesicles **on your eyelid?**
  o a. Yes
  o b. No
32. **Do you experience eye itching?**
  o a. Yes
  o b. No
33. **Do you experience red eye?**
  o a. Yes
  o b. No
34. **Do you experience eye** pain**?**
  o a. Yes
  o b. No
35. **Do you experience excessive tearing (dacryorrhea)?**
  o a. Yes
  o b. No
36. **Do you experience blurred vision?**
  o a. Yes
  o b. No
37. **Do you experience photophobia (light sensitivity)?**
  o a. Yes
  o b. No
38. **Have you been diagnosed** with **a corneal ulcer?**
  o a. Yes
  o b. No
39. **Have you been diagnosed with a chalazion?**
  o a. Yes
  o b. No
40. **Have you been diagnosed with a stye?**
  o a. Yes
  o b. No
41. **Have you been diagnosed with** meibomianitis**?**
  o a. Yes
  o b. No
42. **Do you experience loss of visual** acuity**?**
  o a. Yes
  o b. No
  o c. Do not know
43. **Have you been diagnosed by a doctor with** conjunctivitis**?**
  o a. Allergic conjunctivitis
  o b. Bacterial conjunctivitis
  o c. Viral conjunctivitis
  o d. No
44. **Have you been diagnosed by a doctor** with **keratitis?**
  o a. Bacterial keratitis
  o b. Fungal keratitis
  o c. Viral keratitis
  o d. No

## References

[1] H. Zengin , S. Y. Çaka , E. E. Özdede , İ. T. Tatar , and N. Çınar , “Knowledge, Practices and Use of Contact Lenses Among University Students in Turkey,” Malawi Medical Journal 33, no. 4 (2021): 253–260. 35291383 10.4314/mmj.v33i4.5 PMC8892995

[2] N. Efron and R. M. Pearson , “Centenary Celebration of Fick's Eine Contactbrille,” Archives of Ophthalmology 106, no. 10 (1988): 1370–1377, 10.1001/archopht.1988.01060140534019. 3052382

[3] J. J. Nichols , M. D. P. Willcox , A. J. Bron , et al., “The TFOS International Workshop on Contact Lens Discomfort: Executive Summary,” Investigative Opthalmology & Visual Science 54 (2013): TFOS7–TFOS13. 10.1167/iovs.13-13212 PMC4686219 24058135

[4] Centers for Disease Control and Prevention. (n.d.). About Contact Lens Types. CDC. Retrieved November 24, 2024, from https://www.cdc.gov/contact-lenses/about/about-contact-lens-types.html.

[5] N. K. R. Ibrahim , H. Seraj , R. Khan , M. Baabdullah , and L. Reda , “Prevalence, Habits and Outcomes of Using Contact Lenses Among Medical Students,” Pakistan Journal of Medical Sciences 34, no. 6 (2018): 1429–1434. 30559798 10.12669/pjms.346.16260 PMC6290225

[6] B. Gurnani and K. Kaur , “Contact Lenses. 2023 Jun 11.” StatPearls [Internet] (Treasure Island (FL): StatPearls Publishing, 2024).

[7] J. R. Cope , S. A. Collier , M. M. Rao , et al., “Contact Lens Wearer Demographics and Risk Behaviors for Contact Lens‐Related Eye Infections—United States, 2014,” MMWR. Morbidity and Mortality Weekly Report 64, no. 32 (2015): 865–870. 26292204 10.15585/mmwr.mm6432a2 PMC5779588

[8] K. Edwards , L. Keay , T. Naduvilath , and F. Stapleton , “The Penetrance and Characteristics of Contact Lens Wear in Australia,” Clinical and Experimental Optometry 97, no. 1 (2014): 48–54, 10.1111/cxo.12078. 23834714

[9] B. Mohd‐Ali and X. L. Tan , “Patterns of Use and Knowledge About Contact Lens Wear Amongst Teenagers in Rural Areas in Malaysia,” International Journal of Environmental Research and Public Health 16, no. 24 (2019): 5161, 10.3390/ijerph16245161. 31861174 PMC6950730

[10] N. E. Ezinne , K. K. Ekemiri , G. N. Harbajan , et al., “Contact Lens Prescribing Patterns in a University Clinic in Trinidad and Tobago,” Vision 6, no. 3 (2022): 55, 10.3390/vision6030055. 36136748 PMC9503470

[11] M. Abahussin , M. AlAnazi , K. C. Ogbuehi , and U. L. Osuagwu , “Prevalence, Use and Sale of Contact Lenses in Saudi Arabia: Survey on University Women and Non‐Ophthalmic Stores,” Contact Lens and Anterior Eye 37, no. 3 (2014): 185–190, 10.1016/j.clae.2013.10.001. 24211011

[12] G. Liu , Y. Zhang , E. van der Mark , et al., “Assessing the Origin of Bacteria in Tap Water and Distribution System in an Unchlorinated Drinking Water System by Sourcetracker Using Microbial Community Fingerprints,” Water Research 138 (2018): 86–96, 10.1016/j.watres.2018.03.043. 29573632

[13] F. Stapleton , M. Bakkar , N. Carnt , et al., “BCLA CLEAR—Contact Lens Complications,” Contact Lens and Anterior Eye 44, no. 2 (2021): 330–367, 10.1016/j.clae.2021.02.010. 33775382

[14] M. Guida , V. Di Onofrio , F. Gallè , et al., “Pseudomonas aeruginosa in Swimming Pool Water: Evidences and Perspectives for a New Control Strategy,” International Journal of Environmental Research and Public Health 13, no. 9 (2016): 919, 10.3390/ijerph13090919. 27649225 PMC5036752

[15] J. R. Cope , S. A. Collier , H. Nethercut , J. M. Jones , K. Yates , and J. S. Yoder , “Risk Behaviors for Contact Lens‐Related Eye Infections Among Adults and Adolescents—United States, 2016,” MMWR. Morbidity and Mortality Weekly Report 66, no. 32 (2017): 841–845, 10.15585/mmwr.mm6632a2. 28817556 PMC5657667

[16] K. Sapkota , “Level of Compliance in Contact Lens Wearing Medical Doctors in Nepal,” Contact Lens and Anterior Eye 38, no. 6 (2015): 456–460, 10.1016/j.clae.2015.05.010. 26048663

[17] S. V. Waghmare and S. Jeria , “A Review of Contact Lens‐Related Risk Factors and Complications,” Cureus 14, no. 10 (2022): e30118, 10.7759/cureus.30118. 36381898 PMC9644230

[18] A. Stellwagen , C. MacGregor , R. Kung , A. Konstantopoulos , and P. Hossain , “Personal Hygiene Risk Factors for Contact Lens‐Related Microbial Keratitis,” BMJ Open Ophthalmology 5, no. 1 (2020): e000476, 10.1136/bmjophth-2020-000476. PMC7481083 32953996

[19] O. Albasheer , I. M. Gosadi , I. Abuallut , et al., “Awareness and Hygiene Practices Among Contact Lens Wearers: A Population‐Based Cross‐Sectional Survey,” Cureus 16, no. 2 (2024): e54723, 10.7759/cureus.54723. 38523955 PMC10960920

[20] D. Mingo‐Botín , J. Zamora , F. Arnalich‐Montiel , and F. J. Muñoz‐Negrete , “Characteristics, Behaviors, and Awareness of Contact Lens Wearers Purchasing Lenses Over the Internet,” Eye & Contact Lens: Science & Clinical Practice 46, no. 4 (2020): 208–213, 10.1097/ICL.0000000000000702. 32443017

[21] N. B. Omali , L. N. Subbaraman , M. Heynen , et al., “Lipid Deposition on Contact Lenses in Symptomatic and Asymptomatic Contact Lens Wearers,” Contact Lens and Anterior Eye 44, no. 1 (2021): 56–61, 10.1016/j.clae.2020.05.006. 32466858

[22] L. B. Szczotka‐Flynn , E. Pearlman , and M. Ghannoum , “Microbial Contamination of Contact Lenses, Lens Care Solutions, and Their Accessories: A Literature Review,” Eye & Contact Lens: Science & Clinical Practice 36, no. 2 (2010): 116–129, 10.1097/ICL.0b013e3181d20cae. PMC3482476 20168237

[23] C. McAnally , R. Walters , A. Campolo , et al., “Antimicrobial Efficacy of Contact Lens Solutions Assessed by ISO Standards,” Microorganisms 9, no. 10 (2021): 2173, 10.3390/microorganisms9102173. 34683493 PMC8540466

[24] M. Eltis , “Contact‐Lens‐Related Microbial Keratitis: Case Report and Review,” Journal of Optometry 4, no. 4 (2011): 122–127, 10.1016/S1888-4296(11)70053-X.

[25] D. C. Chang , G. B. Grant , K. O'Donnell , et al., Fusarium Keratitis Investigation Team ., “Multistate Outbreak of Fusarium Keratitis Associated With Use of a Contact Lens Solution,” Journal of the American Medical Association (Chicago, IL) 296, no. 8 (2006): 953–963, 10.1001/jama.296.8.953. 16926355

[26] S. E. Ma , K. So , P. Chung , H. T. Tsang , and S. Chuang , “A Multi‐Country Outbreak of Fungal Keratitis Associated With a Brand of Contact Lens Solution: The Hong Kong Experience,” International Journal of Infectious Diseases 13, no. 4 (2009): 443–448, 10.1016/j.ijid.2007.12.018. 19019715

[27] G. Walther , S. Stasch , K. Kaerger , et al., “Fusarium Keratitis in Germany,” Journal of Clinical Microbiology 55, no. 10 (2017): 2983–2995, 10.1128/JCM.00649-17. 28747368 PMC5625384

